# Polarization Domain Spectrum Sensing Algorithm Based on AlexNet

**DOI:** 10.3390/s22228946

**Published:** 2022-11-18

**Authors:** Shiyu Ren, Hailong Wu, Wantong Chen, Dongxia Li

**Affiliations:** School of Electronic Information and Automation, Civil Aviation University of China, Tianjin 300300, China

**Keywords:** spectrum sensing, polarization information, deep learning, AlexNet

## Abstract

In this paper, we propose a spectrum sensing algorithm based on the Jones vector covariance matrix (JCM) and AlexNet model, i.e., the JCM-AlexNet algorithm, by taking advantage of the different state characteristics of the signal and noise in the polarization domain. We use the AlexNet model, which is good at extracting matrix features, as the classification model and use the Jones vector, which characterizes the polarization state, to calculate its covariance matrix and convert it into an image and then use it as the input to the AlexNet model. Then, we calculate the likelihood ratio test statistic (AlexNet-LRT) based on the output of the model to achieve the classification of the signal and noise. The simulation analysis shows that the JCM-AlexNet algorithm performs better than the conventional polarization detection (PSD) algorithm and the other three (LeNet5, long short-term memory (LSTM), multilayer perceptron (MLP)) excellent deep-learning-based spectrum sensing algorithms for different signal-to-noise ratios and different false alarm probabilities.

## 1. Introduction

Cognitive radio improves spectrum utilization by exploiting spectrum holes in the wireless environment, and one of its core technologies is spectrum sensing [1]. Exploring new techniques for spectrum sensing is an important research challenge. Currently, the vast majority of studies on spectrum sensing are based on a particular feature of the received signal in the time or frequency domain [2], such as the amplitude or phase feature. However, there are few studies on spectrum sensing based on the polarization domain information of the signal. In fact, the Jones vector, which characterizes the signal polarization information, contains the amplitude, phase, and polarization angle features of the signal, so the Jones vector can be used for spectrum sensing to further improve the detection performance [3]. In [2], the authors used an orthogonal dual-polarization antenna to receive the signal and obtain the Jones vector characterizing the signal polarization state and then used the optimum-polarization-based combining (OPBC) technique to achieve spectrum sensing. The proposed OPBC technique improves its sensing performance over the energy detection algorithm at the expense of complexity. In [4], the authors performed spectrum sensing based on the Jones vector using the generalized likelihood ratio test (GLRT) algorithm, which does not require prior knowledge of the signal and noise. In [5], the authors introduced the polarization detection (PSD) algorithm, which has higher detection performance compared to the maximum likelihood estimation detection (MLED) algorithm when the signal and noise are known.

In recent years, more and more experts and scholars have applied deep learning algorithms to spectrum sensing [6,7], but most of the research uses the time domain or spectral data of the signal as the input of the deep learning algorithm. In [8], the authors took the time domain data of the signal as the input of the classical deep learning model multilayer perceptron (MLP) [9], and the results showed that MLP has better detection performance compared to the Naive Bayes algorithm. In [10], the authors used the time domain data of the signal as the input to the long short-term memory (LSTM) model mbox [11], which is good at learning the features of time series data, so that the LSTM model extracts the time-dependent features of the signal. There is a significant improvement in the detection probability at a low signal-to-noise ratio (SNR). In [12], the authors used the time domain data of the signal after preprocessing as the input to the LeNet5 model [13], which is good at learning the features of matrix data, and the results showed that the spectrum sensing using the LeNet5 model has a good sensing performance. In [14,15], the authors used the spectral correlation function features (SCFs) of the signal frequency domain as the input to a convolutional neural network (CNN) model to obtain classification results. This algorithm provides a significant performance improvement over the classical cyclic smooth feature detection (CFD) algorithm.

### 1.1. Motivation

To further improve the performance of spectrum sensing without a priori knowledge of the signal and noise, this paper explores the use of the polarization domain features of signals combined with deep learning algorithms for spectrum sensing. The polarization information of the signal contains both the time domain and frequency domain features, and the polarization information is vectorial and contains the directional information of the signal, so there are more features available for spectrum sensing based on the polarization information of the signal. However, as mentioned earlier, traditional spectrum sensing algorithms based on polarization information have the drawbacks of high complexity [2], or require a priori knowledge [5], or have poor sensing performance [4]. In contrast, deep-learning-based spectrum sensing algorithms often do not require a priori knowledge of the signal and noise, and they have higher detection performance compared to traditional non-intelligent spectrum sensing algorithms [16]. Furthermore, since the input features are one of the determinants of the performance of deep learning algorithms [17] and the polarization information of the signal contains more signal features, we explore the use of deep learning techniques to learn the polarization domain features of the signal and noise, so that a good sensing performance can still be obtained without the need for a priori knowledge of the signal and noise.

### 1.2. Novelty

The novelty of this paper is summarized as follows: (1) In contrast to most current methods that use the time or frequency domain information of the signal for spectrum sensing, we use the polarization domain information of the signal for spectrum sensing. We use the Jones vector to characterize the polarization state of the signal, which contains multiple features of the signal amplitude, phase, and polarization angle at the same time. Therefore, it has a higher detection performance compared to most of the spectrum sensing methods based on one feature of the signal [17]. (2) We are the first to combine polarization domain information with deep learning techniques for spectrum sensing. The JCM AlexNet algorithm first converts the signal into the Jones vector covariance matrix (JCM) and then uses it as the input to the AlexNet algorithm. The algorithm does not need any prior information and can directly learn the features of the JCM, thus improving the spectrum sensing performance. (3) We design the test statistic of the JCM-AlexNet algorithm according to the Neyman–Pearson theorem (NP), so that the proposed algorithm can set the threshold value by the false alarm probability to ensure the rationality of the test statistic design.

The rest of this article is organized as follows. In Section 2, we introduce a Jones vector signal model. Section 3 describes the data preprocessing of the received signals and the online learning and offline detection process of the JCM-AlexNet algorithm. In Section 4, the performance of the JCM-AlexNet algorithm is simulated, verified, and compared with four algorithms, LeNet5, LSTM, MLP, and PSD. The conclusion is discussed in Section 5.

## 2. Polarization Domain Signal Model

The vast majority of spectrum sensing algorithms use only one-dimensional scalar feature in the time or frequency domain of the signal for sensing. In fact, the two-dimensional vector feature of the signal polarization domain is also important information that can be used, including the Jones vector, which features the polarization information of the signal [18,19]. In the following, we will generate the Jones vector signal model.

### 2.1. Jones Vector

According to the classical electromagnetic wave polarization theory [20], the complex vector expression of the electromagnetic wave is:

(1) $$e_{x}\left(t,r\right)=\bar{e}\cdot \exp\left[j\left(\omega t-k^{T}r\right)\right.$$

where $\bar{e}$ is the complex electric field vector, ω is the signal frequency, *r* is the coordinate vector of any point in space, and $k=\frac{2\pi }{\lambda }$ is the propagation vector; if the electromagnetic wave propagates along the +z direction, only the cross-section of z=0 is considered, then the vertical and horizontal components of the complex electric field vector for:

(2) $$\bar{e}=\left[\begin{array}{c} E_{H}\\ E_{V} \end{array}\right]=\left[\begin{array}{c} \left|E_{H}\right|e^{j\phi_{H}}\\ \left|E_{V}\right|e^{j\phi_{V}} \end{array}\right]$$

Therefore, the complex vector expression of the electromagnetic wave can be simplified as:

(3) $$e_{x}\left(t,z\right)=\left[\begin{array}{c} \left|E_{H}\right|e^{j\phi_{H}}e^{j\omega t}\\ \left|E_{V}\right|e^{j\phi_{V}}e^{j\omega t} \end{array}\right]=\left[\begin{array}{c} \left|E_{H}\right|e^{j\phi_{H}}\\ \left|E_{V}\right|e^{j\phi_{V}} \end{array}\right]e^{j\omega t}$$

where $|E_{H}|$ and $|E_{V}|$ represent the horizontal and vertical polarization components of the signal.

However, electromagnetic waves with any single frequency can be decomposed into any two mutually orthogonal polarization states (called polarization bases). In this paper, the commonly used vertical (*V*) and horizontal (*H*) orthogonal polarization bases are used to express:

(4) $$E_{HV}=\left[\begin{array}{c} \left|E_{H}\right|e^{j\phi_{H}}h\\ \left|E_{V}\right|e^{j\phi_{V}}v \end{array}\right]$$

Therefore, the polarization state of the electromagnetic wave can be expressed as the following two-dimensional vector, the Jones vector:

(5) $$E_{\mathrm{Jones}}=E_{A}\left[\begin{array}{c} \cos\gamma \\ \sin\gamma e^{j\phi } \end{array}\right]$$

where $E_{A}$ represents the amplitude of the signal electric field, $E_{A}={\left({\left|E_{H}\right|}^{2}+{\left|E_{V}\right|}^{2}\right)}^{1/2}$, $\left|E_{H}\right|=E_{A}\cos\theta$, $\left|E_{V}\right|=E_{A}\sin\theta$; γ represents the angle of polarization, $\gamma =\arctan\left(E_{V}/E_{H}\right)$, $\gamma \in \left[0,\frac{\pi }{2}\right)$; ϕ represents the phase difference of polarization, $\phi =\phi_{H}-\phi_{V},\phi \in \left[-\pi ,\pi \right)$.

It can be seen from Equation (5) that only three parameters $E_{A},\gamma ,\phi$ can be used to fully represent the trajectory shape of the electric field vector of the plane electromagnetic wave. If only the horizontal and vertical components of the electric field are considered, the normalized Jones vector can be used:

(6) $$E_{\mathrm{Jones}}^{\prime }=\left[\begin{array}{c} \cos\gamma \\ \sin\gamma e^{j\phi } \end{array}\right]$$

### 2.2. Signal Model

Spectrum sensing can be expressed as a binary hypothesis test problem:

(7) $$x\left(n\right)=\left\{\begin{array}{cc} u[n], & H_{0}\\ \bar{s}\left[n\right]+u\left[n\right], & H_{1} \end{array}\;\left(n=1,2,\ldots ,N\right)\right.$$

where $H_{1}$ represents that the channel to be detected is occupied by the user; $H_{0}$ represents that the channel is idle; $x\left[n\right]=\left[\begin{array}{c} x_{H}\left[n\right]\\ x_{V}\left[n\right] \end{array}\right],\;x\left[n\right]≜x\left[nT_{s}\right]∼CN\left(0,R_{x}\right)$ represents a sample of the received signal, $1/T_{s}$ is the sampling rate, $t=nT_{s}$; $u\left[n\right]=\left[\begin{array}{c} u_{H}\left[n\right]\\ u_{V}\left[n\right] \end{array}\right],u\left[n\right]≜u\left[nT_{s}\right]∼CN\left(0,R_{u}\right)$ represents independent and identically distributed two-channel additive Gaussian white noise; $\bar{s}\left[n\right]≜\bar{s}\left[nT_{s}\right]∼CN\left(0,R_{\bar{s}}\right)$ represents a primary user (PU) signal sample after passing through a fading channel. When ignoring the effect of channel multipath delay, $\bar{s}\left[n\right]=\left[\begin{array}{c} {\bar{s}}_{H}\left[n\right]\\ {\bar{s}}_{V}\left[n\right] \end{array}\right]$, $\bar{s}\left[n\right]$ can be expressed as $\bar{s}\left[n\right]=h\left[n\right]s\left[n\right]$, and h[n] is a dual-polarized channel, which can be expressed as:

(8) $$h\left[n\right]=\left(\begin{array}{cc} h_{HH}\left[n\right]e^{j\phi_{1}\left[n\right]} & h_{HV}\left[n\right]e^{j\phi_{2}\left[n\right]}\\ h_{VH}\left[n\right]e^{j\phi_{3}\left[n\right]} & h_{VV}\left[n\right]e^{j\phi_{4}\left(n\right]} \end{array}\right)$$

where $h_{xy}$ represents the channel complex gain between the transmitted polarization component *y* and the received polarization component *x* and $\phi_{i}$ represents its random phase; because s[n] is the transmitted signal sample containing the polarization information Jones vector, it can be expressed as:

(9) $$s\left[n\right]=E_{\mathrm{Jones}\;}^{\prime }e^{j\omega t}=\left[\begin{array}{c} \cos\gamma \\ \sin\gamma e^{j\phi } \end{array}\right]e^{j\omega t}$$

Therefore, the generated signal model is the Jones vector signal model.

## 3. JCM-AlexNet Spectrum Sensing Algorithm

**Figure 1:**
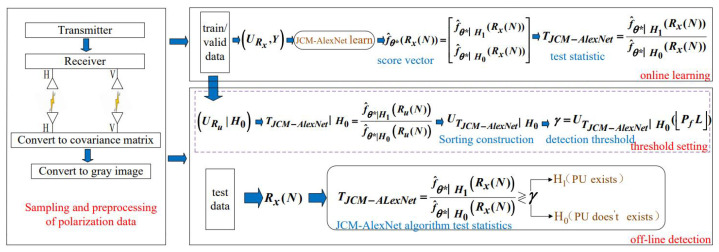
JCM-AlexNet spectrum sensing algorithm framework.

The JCM-AlexNet spectrum sensing algorithm is mainly composed of three parts: data preprocessing, online learning, and offline detection, as shown in Figure 1. It performs spectrum sensing based on the obtained historical polarization information data, without prior information of the PU and the determination of the noise distribution. The proposed algorithm first collects the polarization data of the signal using an orthogonal dual-polarization antenna and converts the polarization data into the JCM after data preprocessing. Then, it is further converted into a grayscale image, and the AlexNet model is used for feature extraction. Finally, according to the learning results of the model, the likelihood ratio test statistics are constructed, and the threshold value is set and detected by using the trained model.

### 3.1. Data Preprocessing of Polarization Signal

Assume that we use *M* orthogonal dual-polarization antennas to receive signals. The received signals can be expressed as:

(10) $$x_{i}\left(n\right)=\left\{\begin{array}{cc} u_{i}\left[n\right], & H_{0}\\ {\bar{s}}_{i}\left[n\right]+u_{i}\left[n\right], & H_{1} \end{array}\;\left(n=1,2,\ldots ,N\right)\left(i=1,2,\ldots ,M\right)\right.$$

where ${\bar{s}}_{i}\left[n\right]=h\left[n\right]s_{i}\left[n\right],s_{i}\left[n\right]=Ae^{j\omega t},A=\left[E_{\mathrm{Jones}1,\;}^{\prime }E_{\mathrm{Jones}\;2,}^{\prime }\cdots E_{\mathrm{Jones}\;M}^{\prime }\right]$ is the polarization domain steering vector matrix.

Let:

(11) $$X={\left[x_{1},x_{2},\cdots ,x_{M}\right]}^{T}$$

(12) $$u={\left[u_{1},u_{2},\cdots ,u_{M}\right]}^{T}$$

$u_{i}$ is the noise received by the *i*-th antenna; $x_{i}$ is the sampled signal received by the *i*-th antenna; the expansion of *X* is written in the following matrix form:

(13) $$X=\left[\begin{array}{c} x_{1}\\ x_{2}\\ \vdots \\ x_{M} \end{array}\right]=\left[\begin{array}{cccc} x_{1}\left(1\right) & x_{1}\left(2\right) & \cdots & x_{1}\left(N\right)\\ x_{2}\left(1\right) & x_{2}\left(2\right) & \cdots & x_{2}\left(N\right)\\ \vdots & \vdots & \vdots & \vdots \\ x_{M}\left(1\right) & x_{M}\left(2\right) & \cdots & x_{M}\left(N\right) \end{array}\right]$$

then the sample covariance matrix of the sampled signal is:

(14) $$R_{x}\left(N\right)=\frac{1}{N}\sum_{k=1}^{M}x_{k}x_{k}^{H}=\frac{1}{N}XX^{H}=\left\{\begin{array}{cc} \sigma_{n}^{2}I_{M}, & H_{0}\\ R_{s}\left(N\right)+\sigma_{n}^{2}I_{M}, & H_{1} \end{array}\right.$$

where $\sigma_{n}^{2}$ is the noise variance, $I_{M}$ is the identity matrix of order *M*, and $R_{s}\left(N\right)$ is the sample covariance matrix of the transmitted signal.

**Figure 2:**
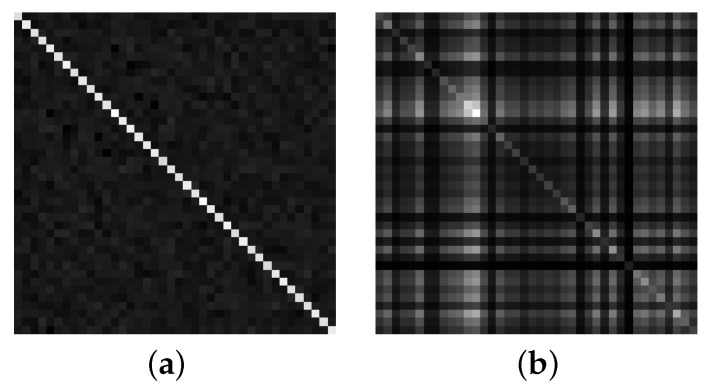
Grayscale images of the sample covariance matrix of the noise and signal with 40 orthogonal dual-polarization antennas: (**a**) $H_{0}$ under $R_{x}\left(N\right)$; (**b**) $H_{1}$ under $R_{x}\left(N\right)$.

Assuming that there are 40 orthogonal dual-polarization antennas, the grayscale images of the sample covariance matrices under the assumptions $H_{0}$ and $H_{1}$ are shown in Figure 2a and Figure 2b, respectively. When there is no PU, the values other than the diagonal are small and the grayscale image brightness distribution is relatively uniform; when there is a PU, the values other than the diagonal are large and the grayscale image brightness distribution is not uniform.

The elements in the covariance matrix represent the correlation between random variables; the smaller the correlation, the smaller the element value and the darker the grayscale image are. The diagonal elements are the autocorrelation of random variables with strong correlation and larger values, and the diagonal brightness of the grayscale image is brighter; the non-diagonal elements indicate intercorrelation between random variables with weaker correlation than the autocorrelation, and the non-diagonal brightness is darker compared with the diagonal brightness of the grayscale image. As shown in Figure 2a, the noise is random in nature; the noise received by different secondary users (SUs) is weakly correlated; the grayscale image beyond the diagonal is darker. As shown in Figure 2b, different SUs receive the same transmit signal, and the intercorrelation between the received useful signals is strong, so the grayscale image brightness is brighter beyond the diagonal compared to Figure 2a.

The grayscale images of the useful signal and the noise are distinctly different, i.e., the covariance matrix features of the useful signal and the noise are distinctly different. Since the AlexNet model is good at learning matrix features, we use it to extract covariance matrix features to achieve spectrum sensing.

### 3.2. Online Learning of JCM-AlexNet Algorithm

The JCM-AlexNet algorithm sends the grayscale image of the labeled JCM sample data to the AlexNet model for learning, so that the AlexNet model learns the appropriate parameters to obtain the two maximum posterior probabilities (MAPs) about the signal and noise and uses them to construct the likelihood ratio test (LRT) to compare with the threshold value to achieve the correct classification of the signal and noise. This section is mainly composed of three parts: first, we introduce the structure of the AlexNet model, then analyze its learning process, and finally, we construct and analyze the LRTS according to the learning results.

#### 3.2.1. AlexNet Model Structure

**Figure 3:**
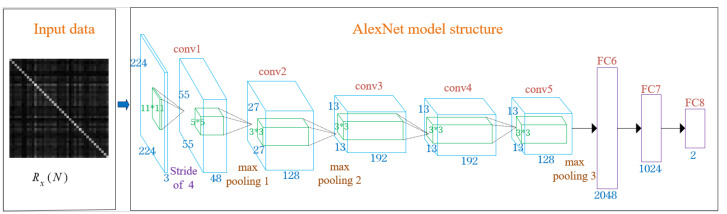
AlexNet model structure.

The internal structure of the AlexNet model is shown in Figure 3. The grayscale images of the JCM is taken as the input data of the model, and the blue part is a convolutional layer; the max pooling layer is used after Convolution Layer 1, Convolution Layer 2, and Convolution Layer 5; the purple part is a fully connected layer; the green part is the convolution kernel; since the dataset in this paper is small, in order to speed up the training, the number of convolution kernels in each convolutional layer is halved; the first convolutional layer uses 48 convolution kernels of 11×11; the second convolutional layer uses 128 convolution kernels of 5×5; the third convolutional layer uses 192 convolution kernels of 3×3; the fourth convolutional layer uses 192 convolution kernels of 3×3; the fifth convolutional layer uses 128 convolution kernels of 3 × 3; finally, three fully connected layers are used for the final classification. Since this paper is a binary hypothesis test problem, therefore, the size of the last fully connected layer is 2.

#### 3.2.2. Learning Phase

During the learning phase, the labeled sample grayscale images are sampled as the training dataset:

(15) $$\left(U_{R_{x}},Y\right)=\left\{\left(R_{x}^{\left(1\right)}\left(N\right),y^{\left(1\right)}\right),\left(R_{x}^{\left(2\right)}\left(N\right),y^{\left(2\right)}\right)\cdots ,\left(R_{x}^{\left(K\right)}\left(N\right),y^{\left(K\right)}\right)\right\}$$

where $U_{R_{x}}$ represents the set of $R_{x}\left(N\right)$, *Y* represents the set of labels *y*, and $\left(R_{x}^{\left(K\right)}\left(N\right),y^{\left(K\right)}\right)$ represents the k−th data of the training dataset (k=123,…,k). In addition, the labels of the training data and validation data are encoded as one-hot encoding:

(16) $$y^{\left(K\right)}=\left\{\begin{array}{c} {\left[1,0\right]}^{T},H_{1}\\ {\left[0,1\right]}^{T},H_{0} \end{array}\right.$$

where $H_{1}$ represents a PU. Correspondingly, the output of the last fully connected layer of the AlexNet model is represented as a 2×1 class score vector:

(17) $$f_{\theta }\left(R_{x}^{\left(K\right)}\left(N\right)\right)=\left[\begin{array}{c} f_{\theta |H_{1}}\left(R_{x}^{\left(K\right)}\left(N\right)\right)\\ f_{\theta |H_{0}}\left(R_{x}^{\left(K\right)}\left(N\right)\right) \end{array}\right]$$

where $f_{\theta }\left(\cdot \right)$ is the expression containing the AlexNet model parameter θ and $f_{\theta |H_{i}}\left(\cdot \right)$ is the expression corresponding to $H_{i}$. In this case, $f_{\theta |H_{i}}\left(R_{x}^{\left(K\right)}\left(N\right)\right)$ represents the classification score of $H_{i}$.

Therefore, we have the following two hypothetical probability expressions:

(18) $$\begin{array}{cc} \; & P\left(y^{\left(k\right)}=1|R_{x}^{\left(K\right)}\left(N\right);\theta \right)=f_{\theta |H_{1}}\left(R_{x}^{\left(K\right)}\left(N\right)\right)\;H_{1}\\ \; & P\left(y^{\left(k\right)}=0|R_{x}^{\left(K\right)}\left(N\right);\theta \right)=f_{\theta |H_{0}}\left(R_{x}^{\left(K\right)}\left(N\right)\right)\;H_{0} \end{array}$$

where $P\left(y^{\left(k\right)}=i|R_{x}^{\left(K\right)}\left(N\right);\theta \right)$ is the conditional probability of $P\left(y^{\left(k\right)}=i|R_{x}^{\left(K\right)}\left(N\right)\right)$ under θ when i=1 or 0.

Therefore, the goal of AlexNet model training is to maximize the conditional probability, namely:

(19) $$L\left(\theta \right)=P\left(Y|U_{R_{x}};\theta \right)=\prod_{k=1}^{K}{\left(f_{\theta |H_{1}}\left(R_{x}^{\left(K\right)}\left(N\right)\right)\right)}^{y^{\left(k\right)}}{\left(f_{\theta |H_{0}}\left(R_{x}^{\left(K\right)}\left(N\right)\right)\right)}^{1-y^{\left(k\right)}}$$

For the convenience of calculation, we introduce the logarithmic function:

(20) $$l\left(\theta \right)=\log L\left(\theta \right)=\sum_{k=1}^{K}y^{\left(k\right)}\log f_{\theta }\left(R_{x}^{\left(K\right)}\left(N\right)\right)+\left(1-y^{\left(k\right)}\right)\log\left(1-f_{\theta }\left(R_{x}^{\left(K\right)}\left(N\right)\right)\right)$$

We want l(θ) to be as large as possible, that is −l(θ) to be as small as possible, and we can obtain the loss function:

(21) $${\mathrm{Loss}}_{JCM-\;\mathrm{AlexNet}\;}\left(\theta \right)=-\frac{1}{K}\sum_{k=1}^{K}y^{\left(k\right)}\log\left({\hat{f}}_{\theta |H_{1}}\left(R_{x}^{\left(k\right)}\left(N\right)\right)\right)+\left(1-y^{\left(k\right)}\right)\log\left({\hat{f}}_{\theta |H_{0}}\left(R_{x}^{\left(k\right)}\left(N\right)\right)\right)$$

The goal of AlexNet training is to obtain the optimal θ to maximize the MAP $P\left(Y|U_{R_{x}}\right)$, namely:

(22) $$\theta^{*}=\arg\max_{\theta }P\left(Y|R_{x}^{\left(k\right)}\left(N\right);\theta \right)$$

where $\theta^{*}$ represents the best θ under the MAP.

As the loss function derived based on Equation (21), we used the Adam optimization algorithm to update the parameter θ of the AlexNet model step by step, so that the training process converges, that is the AlexNet model obtains the best parameter θ, and finally, we obtain the trained AlexNet model, which can be expressed as:

(23) $$f_{\theta^{*}}\left(R_{x}\right)=\left[\begin{array}{c} f_{\theta^{*|}H_{1}}\left(R_{x}\right)\\ f_{\theta^{*|}H_{0}}\left(R_{x}\right) \end{array}\right]$$

where $f_{\theta^{*}}\left(R_{x}\right)$ represents the expression of the AlexNet model trained after inputting $R_{x}$ and $f_{\theta^{*|H_{i}}}\left(R_{x}\right)$ represents the classification score of $H_{i}$.

#### 3.2.3. Test Statistic Design

According to the results of the training process in the previous section, namely Equations (22) and (23), two MAPs can be obtained:

(24) $$\begin{array}{cc} P\left(H_{1}|R_{x}\right)=f_{\theta^{*}|H_{1}}\left(R_{x}\right) & H_{1}\\ P\left(H_{0}|R_{x}\right)=f_{\theta^{*}|H_{0}}\left(R_{x}\right) & H_{0} \end{array}$$

According to Bayesian theorem, we can obtain:

(25) $$P\left(R_{x}|H_{1}\right)=\frac{P\left(H_{1}|R_{x}\right)\cdot P\left(R_{x}\right)}{P\left(H_{1}\right)}=\frac{f_{\theta^{*}|H_{1}}\left(R_{x}\right)\cdot P\left(R_{x}\right)}{P\left(H_{1}\right)}$$

(26) $$P\left(R_{x}|H_{0}\right)=\frac{P\left(H_{0}|R_{x}\right)\cdot P\left(R_{x}\right)}{P\left(H_{0}\right)}=\frac{f_{\theta^{*}|H_{0}}\left(R_{x}\right)\cdot P\left(R_{x}\right)}{P\left(H_{0}\right)}$$

where $P\left(R_{x}|H_{i}\right)$ represents the conditional probability of a given $H_{i}$, $P\left(R_{x}\right)$ is the marginal probability, and $P(H_{i})$ represents the prior probability of $H_{i}$ in the training process. When $P\left(R_{x}|H_{1}\right)$ and $P\left(R_{x}|H_{0}\right)$ are known, the NP theorem proves that the likelihood ratio (LR) has excellent performance as a test statistic.

According to the Neyman–Pearson (NP) theorem, given the false alarm probability $P_{f}=P\left(H_{1}|H_{0}\right)$, in order to maximize the detection probability $P_{d}=P\left(H_{1}|H_{1}\right)$, there are:

(27) $$L\left(R_{x}\right)=\frac{P\left(R_{x}|H_{1}\right)}{P\left(R_{x}|H_{0}\right)}>\lambda$$

where $L\left(R_{x}\right)$ is the likelihood ratio and λ is the detection threshold.

According to the likelihood ratio test (LRT), Equations (25) and (26) are substituted into Equation (27), and we can obtain the AlexNet LRT:

(28) $$L_{\mathrm{AlexNet}\;}\left(R_{x}\right)=\frac{f_{\theta^{*}|H_{1}}\left(R_{x}\right)}{f_{\theta^{*}|H_{0}}\left(R_{x}\right)}\cdot \frac{P\left(H_{0}\right)}{P\left(H_{1}\right)}=\frac{f_{\theta^{*}|H_{1}}\left(R_{x}\right)}{f_{\theta^{*}|H_{0}}\left(R_{x}\right)}=T_{JCM-\;\mathrm{AlexNet}\;}>\lambda$$

where the threshold λ can be obtained from the false alarm probability constraint. For the convenience of analysis, we make the training datasets $H_{1}$ and $H_{0}$ have the same number of samples, that is $P\left(H_{1}\right)=P\left(H_{0}\right)=0.5$; in addition:

(29) $$f_{\theta^{*}|H_{i}}\left(R_{x}\right)=e_{H_{i}}\cdot f_{\theta^{*}}\left(R_{x}\right)$$

(30) $$e_{H_{i}}=\left\{\begin{array}{cc} {}[1,0], & i=1\\ {}[0,1], & i=0 \end{array}\right.$$

### 3.3. Off-Line Detection of JCM-AlexNet Algorithm

The JCM-AlexNet spectrum sensing algorithm is used to classify signals and noise by handing over the grayscale image of unlabeled JCM sample data to a trained model (i.e., the model has been trained to acquire the appropriate parameters) for detection. This section is mainly composed of two parts: firstly, the detection threshold is designed according to the trained model, and then, the probability value obtained from the LRT in the detection process is compared with the threshold to determine whether the PU exists or not.

#### 3.3.1. Design of Detection Threshold

The threshold is designed to send the grayscale images of the noise samples to the trained AlexNet model for detection and to set the false alarm probability according to the requirements, so as to obtain the appropriate threshold. If:

(31) $$R_{x}=R_{u}\left(N\right)=\frac{1}{N}\sum_{n=0}^{N-1}u\left(n\right)u^{H}\left(n\right)$$

where $R_{u}\left(N\right)$ is the sample covariance matrix composed of *N* noise vectors, the expression of $T_{JCM-AlexNet}$ at $H_{0}$ can be obtained:

(32) $$T_{JCM-\;\mathrm{AlexNet}\;}|H_{0}=\frac{{\hat{f}}_{\theta^{*}|H_{1}}\left(R_{u}\left(N\right)\right)}{{\hat{f}}_{\theta^{*}|H_{0}}\left(R_{u}\left(N\right)\right)}$$

According to $P_{f}=P\left(H_{1}|H_{0}\right)$, the false alarm probability can be rewritten as:

(33) $$P_{f}=P\left\{T_{JCM-\;\mathrm{AlexNet}\;}|H_{0}>\lambda \right\}$$

Let $U_{R_{u}}=\left\{R_{u}^{\left(1\right)}\left(N\right),R_{u}^{\left(2\right)}\left(N\right),\cdots ,R_{u}^{\left(L\right)}|\left(N\right)\right\}$ represent the dataset under $H_{0}$, which is composed of *L* data; send them to the trained JCM-AlexNet model; obtain all the $T_{JCM-\;\mathrm{AlexNet}\;}|H_{0}$ values; sort them in descending order; build the $T_{JCM-\;\mathrm{AlexNet}\;}|H_{0}$ set, which is represented as $U_{T_{JCM-\;\mathrm{AlexNet}}|H_{0}}$. The detection threshold can be obtained by:

(34) $$\lambda =U_{T_{JCM-\;\mathrm{AlexNet}}|H_{0}}\left(\left\lfloor P_{f}L\right\rfloor \right)$$

where ⌊.⌋ represents rounding down to the nearest integer and $U_{T_{JCM-\;\mathrm{AlexNet}\;}|H_{0}}\left(l\right)$ represents the value of $U_{T_{JCM-\;\mathrm{AlexNet}\;}|H_{0}}$ of the l−th element.

#### 3.3.2. Determination of Detection Results

In the model detection phase, collect the grayscale images of samples without labeling as test data, represented as ${\tilde{R}}_{x}$; ${\tilde{R}}_{x}$ is transmitted to the trained AlexNet model, and then, the likelihood ratio test statistic (AlexNet-LRTS) detection is performed based on unlabeled samples, because the MAP expression obtained in the training process assumes that the model parameters are unknown, while the detection process calculates the detection probability for the known model parameters; therefore, $P\left(H_{i}|R_{x}\right)$ is not suitable for detection instances, and it is converted into conditional probabilities $P\left(R_{x}|H_{i}\right)$ to derive the LRT in the detection process.

(35) $$L_{JCM-\;\mathrm{AlexNet}\;}\left({\tilde{R}}_{x}\right)=\frac{P\left({\tilde{R}}_{x}|H_{1}\right)}{P\left({\tilde{R}}_{x}|H_{0}\right)}\underset{H_{1}}{\overset{H_{0}}{≶}}\;\lambda$$

where:

(36) $$P\left({\tilde{R}}_{x}|H_{1}\right)=\frac{P\left(H_{1}|{\tilde{R}}_{x}\right)\cdot P\left({\tilde{R}}_{x}\right)}{P\left(H_{1}\right)}=\frac{f_{\theta^{*}|H_{1}}\left({\tilde{R}}_{x}\right)\cdot P\left({\tilde{R}}_{x}\right)}{P\left(H_{1}\right)}$$

(37) $$P\left({\tilde{R}}_{x}|H_{0}\right)=\frac{P\left(H_{0}|{\tilde{R}}_{x}\right)\cdot P\left({\tilde{R}}_{x}\right)}{P\left(H_{0}\right)}=\frac{f_{\theta^{*}|H_{0}}\left({\tilde{R}}_{x}\right)\cdot P\left({\tilde{R}}_{x}\right)}{P\left(H_{0}\right)}$$

Substitute Equations (36) and (37) into Equation (35) to obtain:

(38) $$L_{JCM-\;\mathrm{AlexNet}\;}\left({\tilde{R}}_{x}\right)=\frac{f_{\theta^{*}|H_{1}}\left({\tilde{R}}_{x}\right)}{f_{\theta^{*}|H_{0}}\left({\tilde{R}}_{x}\right)}=T_{JCM-\;\mathrm{AlexNet}\;}\underset{H_{1}}{\overset{H_{0}}{≶}}\;\lambda$$

Therefore, after the test statistics are obtained in the detection phase, they can be compared with the preset threshold, so as to quickly judge whether the PU exists or not.

## 4. Simulation Analysis

We used the Python simulation platform to generate the datasets. First, a series of random sequences was generated at the transmitter side, which were modulated by QPSK and then fed into a Gaussian white noise channel. Then, we used an orthogonal dual-polarization antenna model to receive the polarization information of the signal, and the ratio of the received signal power to the Gaussian white noise power was used as the SNR.

We first analyze the network structure of the proposed JCM-AlexNet algorithm, introduce the role of each layer in the structure, and demonstrate its advantages. Secondly, the hyperparameters and function combinations of JCM-AlexNet, LeNet5, LSTM, and MLP are analyzed, and the hyperparameters and function combinations with the highest detection probability of the four algorithms are obtained, respectively, to ensure the fairness of the algorithm comparison. Then, we compare and analyze the detection probability of the proposed JCM-AlexNet spectrum sensing algorithm with the traditional PSD algorithm and compare and analyze the detection probability with the other three excellent spectrum sensing algorithms based on deep learning (LeNet5, LSTM, MLP). Finally, the change of the loss value in the training process of the proposed algorithm is analyzed, which verifies that it has excellent sensing performance. In the above simulations of the four algorithms, JCM-AlexNet, LeNet5, LSTM, and MLP, we used a total of 2000 matrix data, of which 1700 matrix data were used for the learning phase and the other 300 matrix data were used for the detection phase. In addition, 1500 matrix data were used for training, and 200 matrix data were used for validation in the learning phase.

### 4.1. Structural Analysis

The AlexNet model includes five convolutional layers (conv2d) and three fully connected layers; the convolutional layers are used to extract the features of the JCM; the fully connected layer maps the learned features to the space of sample labels and plays the role of the classifier, as shown in Table 1; because spectrum sensing can be expressed as a binary hypothesis testing problem, the output of the last fully connected layer is two. The ReLU activation function or max pooling layer is also used after the convolutional layer and the fully connected layer; the ReLU activation function turns the output into a non-linear combination of the input, so that the input data can approach any function through model training; the max pooling layer reduces the feature dimension and removes redundant information, thus reducing the number of parameters and simplifying the network complexity; the AlexNet model uses overlapping based on the max pooling layer, which expands the output of the pooling layer into multi-level smaller features, and adopts sparse coding to fuse multi-level features, thus reducing the feature dimension of the pooling layer output. In addition, a dropout layer is used, that is, in the training process of the deep learning network, neural network elements are temporarily discarded from the network according to a certain probability; dropout has a very good effect on preventing overfitting, reducing the parameters of the model, and enhancing the generalization ability of the model.

### 4.2. Parameter Analysis

Choosing different hyperparameters, different loss functions and optimization functions in deep learning will obtain different results. Hyperparameters are variables determined according to the effect of experience and the validation dataset on the model, such as epoch, batch_size, and learning rate. Therefore, we debugged the parameters of each of the four algorithms (JCM-AlexNet, LeNet5, LSTM, and MLP) through simulation, and we took the model with the highest detection probability, respectively, to ensure the fairness of the algorithm comparison. As shown in Table 2. Different loss functions and optimization functions will also affect the final performance of the algorithm; however, since both the binary_crossentropy and categorical_crossentropy loss functions can be used to deal with binary classification tasks, the difference is that the categorical_crossentropy loss function can also be used for multi-classification tasks, so there is almost no difference in choosing the categorical_crossentropy or binary_crossentropy loss functions in this paper. However, choosing different optimizers has a great influence on the detection probability, because, when the optimizer is SGD, the gradient updates frequently, which leads to serious oscillation of the loss function and eventually stays at the local minimum or saddle point, resulting in a decrease in the detection probability. However, the RMSProp optimizer adds second-order momentum on the basis of SGD and uses the sliding window weighted average to calculate the second-order momentum, which solves the problems of the local minimum and saddle point. In addition, the Adam optimizer integrates the first-order momentum of SGD and the second-order momentum of RMSProp. The first-order momentum can reduce the parameter update speed, thus reducing oscillations, and can accelerate the parameter update when the gradients are in the same direction, thus accelerating convergence. The second-order momentum can solve the problem of the local minimum and saddle point. The Adam optimizer, on the other hand, integrates the advantages of the first-order momentum and second-order momentum and is highly robust to the choice of the hyperparameters. Therefore, a higher detection probability is achieved using the Adam optimizer.

In summary, through simulation experiments, we obtained the parameter combination that made the detection probability of the four algorithms (MLP, LSTM, LeNet5, and JCM-AlexNet) the highest, respectively, so as to ensure the fairness of the comparison.

### 4.3. Performance Analysis

This section firstly compares the detection probabilities of JCM-AlexNet, LeNet5, LSTM, MLP, and PSD with different SNRs and false alarm probabilities, then analyzes the change of the loss value of the JCM-AlexNet algorithm with different epochs and different SNRs, and finally, concludes that the proposed JCM-AlexNet algorithm has better performance.

#### 4.3.1. Performance Analysis of Detection Probability

**Figure 4:**
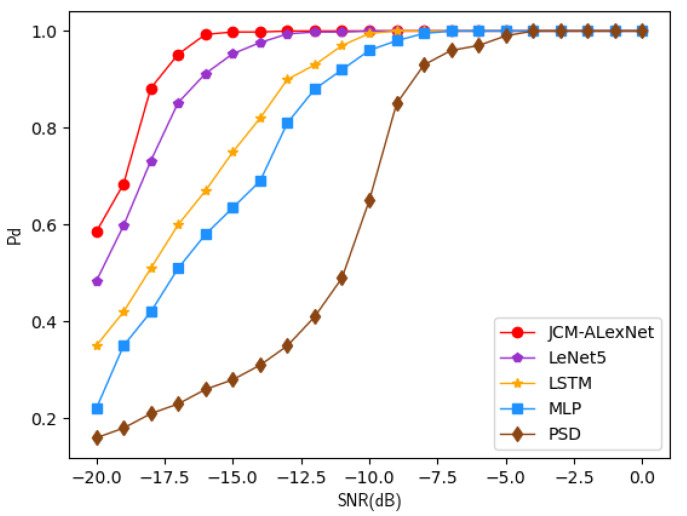
Pd of five algorithms with different SNRs at Pf = 0. 01.

A. Performance analysis of detection probability under different SNRs:

In order to evaluate the performance of the proposed algorithm, when the false alarm probability is 0.01, we compared the detection probabilities of PSD, MLP, LSTM, LeNet5, and JCM-AlexNet in the SNR range of [−20 dB, 0 dB], as shown in Figure 4. It can be seen from the figure that all five algorithms have a high detection probability at a high SNR. Moreover, the proposed JCM-AlexNet algorithm has the highest detection probability at a low SNR. For example, when the SNR is −15 dB, the detection probability of the JCM-AlexNet algorithm is 99.8%, while the detection probabilities of PSD, MLP, LSTM, and LeNet5 are 28.2%, 63.5%, 75.2%, and 95.3%, respectively. This is due to the fact that our algorithm learns the Jones vector containing multiple features of the signal amplitude, phase, and polarization angle, thus allowing more signal features to be learned. In addition, the AlexNet algorithm is good at extracting the features from the matrix data. Therefore, the JCM-AlexNet algorithm has the highest detection probability compared with the other four algorithms.

**Figure 5:**
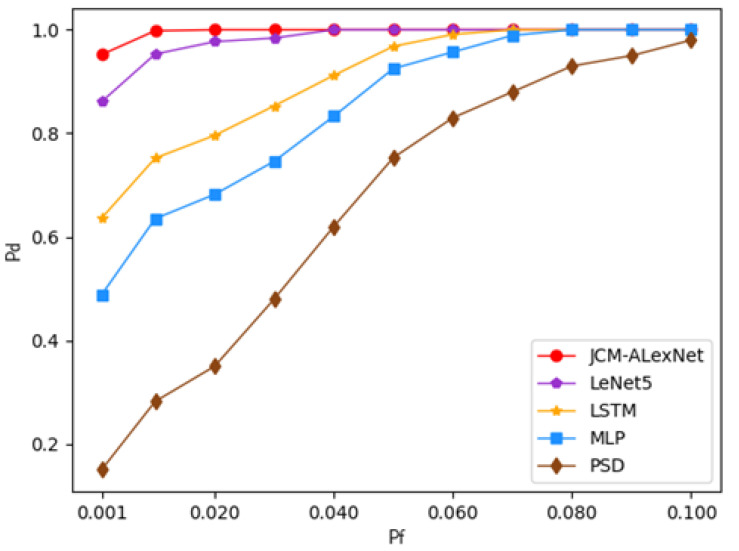
Pd of five algorithms under different Pf when SNR = −15 dB.

B. Performance analysis of detection probability under different false alarm probabilities:

In order to further evaluate the performance of the proposed algorithm, we compared the detection probabilities of PSD, MLP, LSTM, LeNet5, and JCM-AlexNet in the false alarm probability range of (0, 0.1] at SNR = −15 dB, as shown in Figure 5. As we can see from the picture, when the false alarm probability is high, the JCM-AlexNet and LeNet5 algorithms have higher detection probabilities than the other three algorithms. For example, when the false alarm probability is 0.05, the detection probability can reach 100%, while the detection probabilities of PSD, MLP, and LSTM are 75.3%, 92.5%, and 96.8%, respectively. When the false alarm probability is low, our JCM-AlexNet algorithm has the highest detection probability compared with the other four algorithms. For example, when the false alarm probability is 0.01, the detection probability of JCM-AlexNet can reach 99.8%, while the detection probabilities of PSD, MLP, LSTM, and LeNet5 are 28.2%, 63.5%, 75.2%, and 95.3%, respectively. As mentioned earlier, the AlexNet and LeNet5 algorithms are good at feature extraction from the matrix data, so they have a higher detection probability than the other three algorithms. Moreover, the AlexNet algorithm incorporates a dropout layer after the fully connected layer, which can reduce the number of parameters when having a deeper network layer, thus preventing overfitting. In addition, AlexNet uses the ReLU activation function instead of the Sigmoid activation function to optimize the nonlinearity of the input, which can speed up the computation of the model. However, since the ReLU activation function is an asymmetric and nonlinear activation function, it may cause the problem of gradient explosion or disappearance when calculating the gradient. Therefore, we used the Kaiming initialization method, which is specialized to combat this problem, to initialize the weights in the model randomly, so that the proposed JCM-AlexNet algorithm can converge quickly during optimization and avoid falling into local minima, thus further improving the algorithm’s detection performance.

#### 4.3.2. Analysis of Loss Value Change

In deep learning, the loss function is used to evaluate the deviation between the predicted and actual values of the model. The smaller the loss value is, the better the model performance is. Therefore, in the following, we will analyze the variation of the loss value of the JCM-AlexNet algorithm to verify its performance.

A. Analysis of loss value change under different epochs:

In deep learning, an epoch represents all the data input to the model, completing a forward and backward propagation. As the number of epochs increases, the number of iterations to update the model weights increases, and the curve changes from an unfit state at the beginning to an optimally fit state. Therefore, we trained the AlexNet model at epochs of 50, 100, and 200 and found the best epoch by plotting the loss–epoch curve.

**Figure 6:**
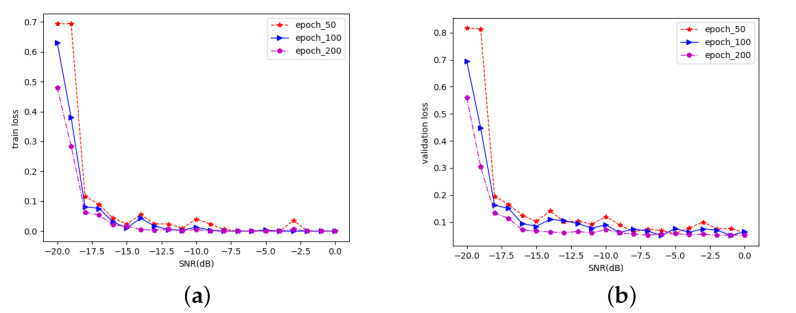
Change of AlexNet model loss value under different epochs. (**a**) Training loss; (**b**) validation loss.

It can be seen from Figure 6 that, at a high SNR, the loss values for epochs of 50, 100, and 200 are all small, i.e., they all result in a good fit of the AlexNet model. However, at a low SNR, the loss values at epoch = 200 are smaller than those at epoch = 50 and 100, so it can be concluded that the AlexNet model can fit the data better at epoch = 200.

B. Analysis of loss value change under different SNRs:

From the previous analysis, it is clear that the AlexNet model can fit the data better at 200 epochs, so we analyzed the change of the loss values of the JCM-AlexNet algorithm at 200 epochs with different SNRs.

**Figure 7:**
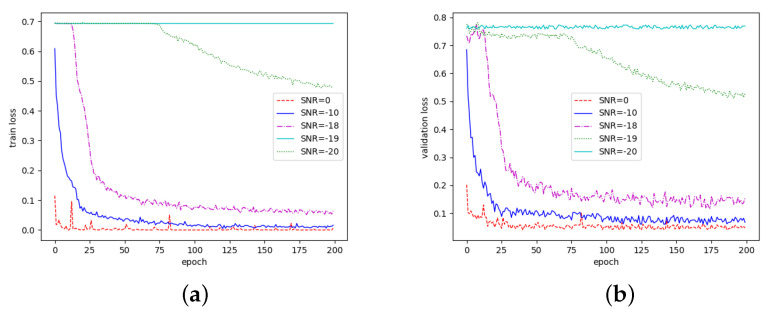
Change of loss value under different SNRs. (**a**) Training loss; (**b**) validation loss.

As shown in Figure 7, firstly, when the SNR is 0dB, the loss value is small and fluctuates little, which indicates that the model can correctly classify the signal and noise in the initial training stage. Secondly, when the SNR is −10 dB and −18 dB, the loss value keeps decreasing in the initial training stage, which indicates that the model cannot classify the signal and noise well at this time. However, with the continuous optimization of the parameters during the training process, the signals and noises can be correctly classified eventually. Thirdly, at an SNR of −19 dB, the loss value decreases continuously during the training process, but fails to reach the minimum value, so the model finally fails to classify the signal and noise correctly. In addition, at an SNR of −20 dB, the loss value is always large and almost unchanged; thus, the model cannot classify the signal and noise. The above analysis shows that our proposed algorithm has better performance at SNRs greater than −18 dB.

## 5. Conclusions

In this paper, the JCM-AlexNet spectrum sensing algorithm was proposed by using the AlexNet model to classify the signal and noise based on the different features of their Jones vector covariance matrix. The algorithm can extract the signal amplitude, phase, and polarization angle features contained in the Jones vector without prior knowledge of the signal and noise. Finally, the JCM-AlexNet algorithm was compared with LeNet5, LSTM, the MLP-based algorithm, and the PSD algorithm, and the simulation results showed that the proposed algorithm has better performance under different SNRs and different false alarm probabilities.

## Figures and Tables

**Table 1:** JCM-AlexNet model structure and corresponding training parameters.

Layer (Type)	Output Shape	Param
Conv2d-1	[−1, 48, 55, 55]	17472
ReLU-2	[−1, 48, 55, 55]	0
MaxPool2d-3	[−1, 48, 27, 27]	0
Conv2d-4	[−1, 128, 27, 27]	153728
ReLU-5	[−1, 128, 27, 27]	0
MaxPool2d-6	[−1, 128, 13, 13]	0
Conv2d-7	[−1, 192, 13, 13]	221376
ReLU-8	[−1, 192, 13, 13]	0
Conv2d-9	[−1, 192, 13, 13]	331968
ReLU-10	[−1, 192, 13, 13]	0
Conv2d-11	[−1, 128, 13, 13]	221312
ReLU-12	[−1, 128, 13, 13]	0
MaxPool2d-13	[−1, 128, 6, 6]	0
Dropout-14	[−1, 4608]	0
Linear-15	[−1, 2048]	9439232
ReLU-16	[−1, 2048]	0
Dropout-17	[−1, 2048]	0
Linear-18	[−1, 1024]	2098176
ReLU-19	[−1, 1024]	0
Linear-20	[−1, 2]	2050

**Table 2:** Detection probability of model parameters and correlation functions when SNR = −15 dB.

	Parameter Value	AlexNet_Pd	LeNet5_Pd	LSTM_Pd	MLP_Pd
Learning rate	$1\times 10^{-3}$	0.952	0.894	0.724	0.589
	$2\times 10^{-3}$	0.998	0.923	0.752	0.643
	$5\times 10^{-3}$	0.973	0.856	0.681	0.635
Batch_size	32	0.981	0.905	0.736	0.614
	64	0.998	0.923	0.752	0.635
epoch	50	0.984	0.916	0.732	0.635
	100	0.992	0.923	0.752	0.602
	200	0.998	0.895	0.744	0.608
loss function	binary_crossentropy	0.995	0.923	0.749	0.635
	categorical_crossentropy	0.998	0.921	0.752	0.635
Optimization function	SGD	0.856	0.828	0.724	0.596
	Adam	0.998	0.923	0.752	0.635
	Rmsprop	0.926	0.854	0.738	0.614

## Data Availability

Not applicable.
